# Development of Smart-Ring-Based Chest Compression Depth Feedback Device for High Quality Chest Compressions: A Proof-of-Concept Study

**DOI:** 10.3390/bios11020035

**Published:** 2021-01-28

**Authors:** Seungjae Lee, Yeongtak Song, Jongshill Lee, Jaehoon Oh, Tae Ho Lim, Chiwon Ahn, In Young Kim

**Affiliations:** 1 Department of Biomedical Engineering, Hanyang University, Seoul 04763, Korea; seungjaelee@hanyang.ac.kr (S.L.); netlee@hanyang.ac.kr (J.L.); 2 Department of Emergency Medicine, College of Medicine, Hanyang University, Seoul 04763, Korea; yeongtaksong@hanyang.ac.kr (Y.S.); ojjai@hanyang.ac.kr (J.O.); erthim@hanyang.ac.kr (T.H.L.); 3 Convergence Technology Center for Disaster Preparedness, Hanyang University, Seoul 04763, Korea; 4 Department of Emergency Medicine, College of Medicine, Chung-Ang University, Seoul 06974, Korea; cahn@cau.ac.kr

**Keywords:** cardiopulmonary resuscitation, inertial measurement units, mobile health, smart ring, chest compression

## Abstract

Recently, a smart-device-based chest compression depth (CCD) feedback system that helps ensure that chest compressions have adequate depth during cardiopulmonary resuscitation (CPR) was developed. However, no CCD feedback device has been developed for infants, and many feedback systems are inconvenient to use. In this paper, we report the development of a smart-ring-based CCD feedback device for CPR based on an inertial measurement unit, and propose a high-quality chest compression depth estimation algorithm that considers the orientation of the device. The performance of the proposed feedback system was evaluated by comparing it with a linear variable differential transformer in three CPR situations. The experimental results showed compression depth errors of 2.0 ± 1.1, 2.2 ± 0.9, and 1.4 ± 1.1 mm in the three situations. In addition, we conducted a pilot test with an adult/infant mannequin. The results of the experiments show that the proposed smart-ring-based CCD feedback system is applicable to various chest compression methods based on real CPR situations.

## 1. Introduction

Cardiopulmonary resuscitation (CPR) is an emergency procedure for manually preserving brain function until further measures can be taken to restore spontaneous blood circulation and breathing in a person in cardiac arrest. According to the 2015 American Heart Association and the European Resuscitation Council Guidelines for Resuscitation 2015, high-quality CPR includes chest compressions of adequate rate and depth, allowing full chest recoil between compressions, minimizing interruptions in chest compressions, and avoiding excessive ventilation [1,2].

For high-quality CPR, healthcare workers and emergency medical staff periodically receive basic life-support education. In addition, various CPR feedback devices have been developed and used [3]. These devices measure chest compression depth (CCD) based on an accelerometer, a pressure sensor, or a combination of two sensors. Several studies have shown that feedback devices improve CCD in simulated cardiac arrest [4,5,6]. Furthermore, according to recent CPR guidelines, it is reasonable to use audiovisual feedback devices during CPR for real-time performance optimization [7].

However, a chest compression (CC) feedback device is not always available. Thus, smartphone-based CPR feedback apps have been developed to overcome this limitation [8,9,10]. These feedback apps provide responses by calculating the CCD and CC rate (CCR) using a built-in accelerometer in the smartphone. Simulation studies have reported that feedback on CPR depth and rate using a smartphone application can help maintain adequate chest compression depth during prolonged CPR. However, holding the smartphone during CPR hampers chest compression [8]. Generally, a smartphone used as a CC feedback device is sandwiched between the two hands, which can lead to inaccurate CCD feedback because of unnecessary movement.

Recently, in response to the disadvantages of the use of smartphone devices, smartwatch-based CC feedback devices have been developed and reported [11,12,13]. Our previous study showed that smartwatch-based CCD feedback was more accurate than smartphone-based feedback [11]. However, it is inconvenient to observe feedback from a smartwatch while performing CPR. In addition, the CPR provider’s wrist is distant from the patient’s sternum, which can reduce accuracy.

There are studies on wearable CPR CCD feedback systems for infants. Dellimore et al. proposed a method to measure CPR depth by attaching an acceleration sensor to a fingernail. However, this wearable device was manufactured in the form of a glove-shaped prototype that was difficult to use [14]. Lee J. et al. suggested a two-finger infant CPR feedback system using a smartwatch. In the two-thumb method, because the thumbs provide depth information, a smartwatch worn on the wrist cannot be used [15].

In our study, we developed a ring-type wearable system based on inertial measurement units (IMUs) to provide depth information during CPR. We proposed a novel CCD estimation algorithm that considers the orientation of the device, obtained using a three-axis gyroscope and a three-axis accelerometer. Performance evaluation was undertaken for three configurations of CPR. In addition, to compare the performance of the proposed ring system to a smartwatch system, an evaluation was performed on a mannequin by four emergency medical professionals who had completed CPR training and had experience with CPR for adults and infants.

## 2. Methods

### 2.1. Hardware System of the CPR Smart Ring

**Figure 1:**
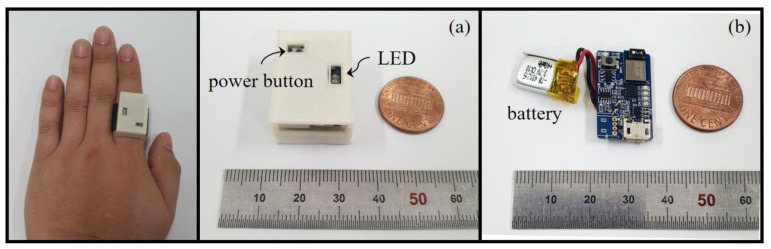
Smart-ring-based chest compression feedback device. (**a**) Overall CPR Smart Ring. (**b**) Electronic circuit board of the CPR Smart Ring.

We developed a ring-type device, called the CPR Smart Ring, to provide feedback on CCD, as shown in Figure 1. This smart-ring-based CCD feedback system was designed for user comfort when performing CPR. The overall CPR Smart Ring dimensions were 20 × 30 × 10 mm^3^ (width, depth, height) with a weight of 6.15 g. The electronic circuit board of the CPR Smart Ring weighed 2.55 g and measured 15 × 25 × 5 mm^3^. Figure 1b shows the size of the electronic circuit board in relation to a one-cent United States coin.

The ring system includes a battery (3.7 V, 40 mAh), a nine-axis (three-axis accelerometer, three-axis gyroscope, three-axis magnetometer) IMU sensor (MPU9250, InvenSense, San Jose, CA, USA), and an integrated module (UTO-NBL-52A, Utovertek, Seongnam, Republic of Korea) that combines a microprocessor (ARM M4 Cortex) with a Bluetooth module (nRF52832, Nordic Semiconductor, Trondheim, Norway). The microprocessor uses IMU data to calculate Euler angles (yaw (ψ), pitch (θ), and roll (ϕ)) and computes the CCD estimation algorithms, which are described in the next paragraph.

### 2.2. Chest Compression Depth Estimation Algorithm

**Figure 2:**
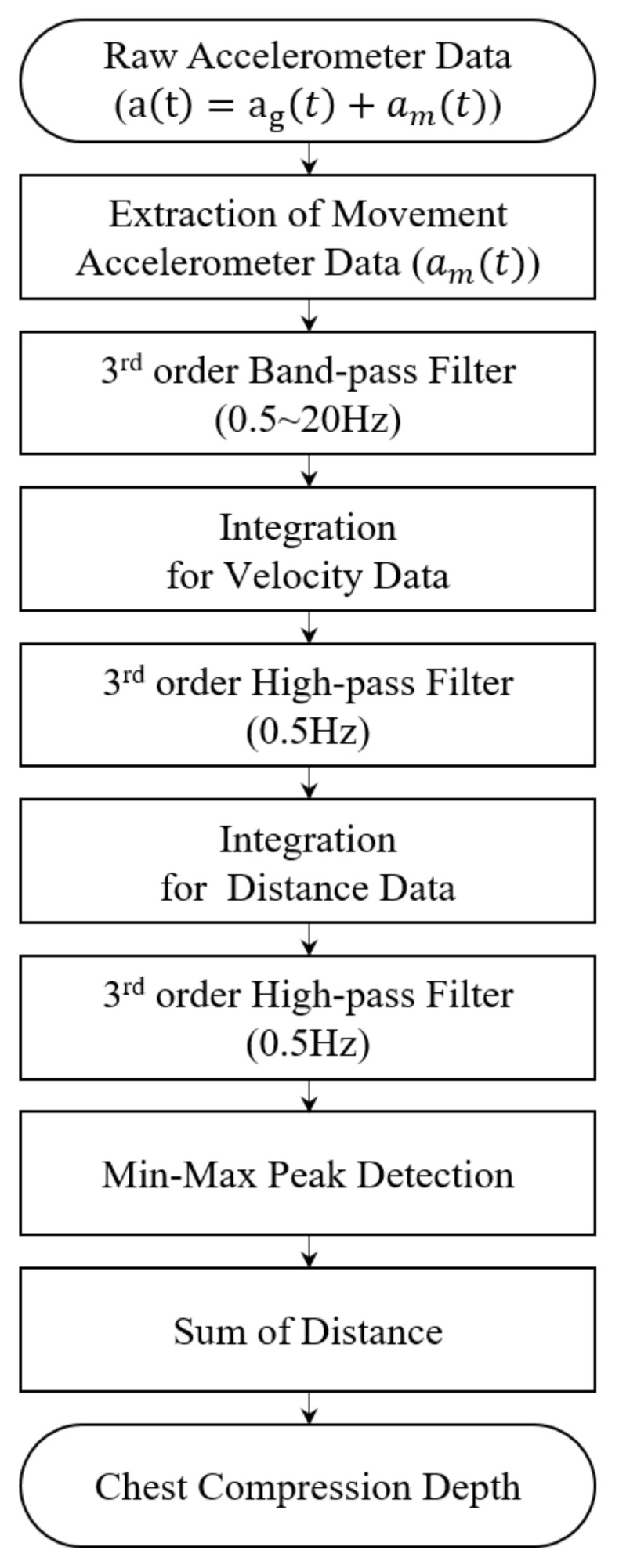
Flow chart of the chest compression depth estimation algorithm.

The flow chart for construction of the CCD estimation algorithm is shown in Figure 2. The algorithm uses raw acceleration with a high-pass filter to remove the effects of gravity acceleration and double integration to calculate CCD.

#### 2.2.1. Extraction of the Movement Component in an Acceleration Signal

The three-axis accelerometer of the IMU sensor, which is built into the ring module, provides acceleration for each axis (x, y, and z). The acceleration value (*a*(*t*)) provided by the three-axis accelerometer consists of the sum of the acceleration due to gravity (*a_g_*(*t*)) and movement (*a_m_*(*t*)), as shown in Equation (1).

(1) $$a\left(t\right)=a_{g}\left(t\right)+a_{m}\left(t\right)$$

**Figure 3:**
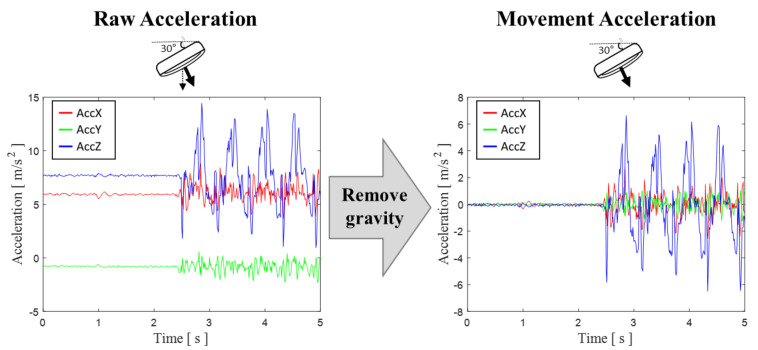
Removing the gravity component from the acceleration signal.

To extract *a_m_*(*t*) by chest compression from an acceleration signal, *a_g_*(*t*) needs to be removed. Because *a_g_*(*t*) changes depending on inclination of the acceleration sensor, the orientation of the sensor must be known. We obtained sensor orientation with a gradient descent algorithm from Madwick et al. using the three-axis acceleration, three-axis angular rate, and three-axis magnetometer information of the IMU [15].The orientation of the sensor is expressed as Euler angles, ψ, θ, and ϕ. By using the Euler angle (θ, ϕ), the orientation of the sensor can be converted into a rotation matrix ($R_{\theta },\;R_{\phi }$) that is easy to mathematically calculate. The total rotation matrix ($R_{total}$) is calculated as shown in Equation (2). Real-time gravity acceleration is obtained using the rotation matrix by setting the z-axis of acceleration parallel to gravity (Equation (3)). The movement acceleration of the CC in real-time can be obtained by removing the gravity component from Equation (1). Figure 3 shows exclusion of the acceleration of gravity to leave only *a_m_*(*t*).

(2) $$R_{total}=R_{\theta }*R_{\phi }\;(R_{\theta }=\left[\begin{array}{ccc} \cos\theta & 0 & -\sin\theta \\ 0 & 1 & 0\\ \sin\theta & 0 & \cos\theta \end{array}\right],\;R_{\phi }=\left[\begin{array}{ccc} 1 & 0 & 0\\ 0 & \cos\phi & \sin\phi \\ 0 & \sin\phi & \cos\phi \end{array}\right])$$

(3) $$a_{g0}=\left(0,0,\;1g\right),\;a_{g}\left(t\right)=a_{g0}*R_{total}$$

#### 2.2.2. CCD Estimation Using a Three-Axis Accelerometer

Displacement during chest compression using an acceleration signal without *a_g_*(*t*) is calculated with double integration, as shown in Equation (4).

(4) $$d\left(t\right)=\int v\left(t\right)dt+C_{d}=\iint a\left(t\right)dtdt+C_{v}+C_{d}$$

Applied to each of the x, y, and z axes, the final distance of motion is the vector sum of the displacement of the three axes. Using the integral constants $C_{v}$ and $C_{d}$ in Equation (4), the distance increases with time. In addition, basic noise is produced based on the characteristics of the accelerometer. To eliminate these noise sources, we designed a Butterworth third-order high-pass filter (HPF) and a third-order low-pass filter (LPF), as in a previous study that used such filters to emphasize weighted smoothing and transient components [10]. In the algorithm by Song et al., it was difficult to acquire acceleration signals periodically using a smartphone based on Android OS, so a conventional frequency selection filter could not be used. To remove the noise, they applied the weighted smoothing technique, which has the role of a low-pass filter. To remove drift, they used the transient components emphasizing technique, which has the function of a high-pass filter. During chest compression, the frequency band of the acceleration signal was 1–10 Hz [16,17]. Therefore, an HPF with 0.5 Hz cut-off frequency was applied to remove the DC component.

**Figure 4:**
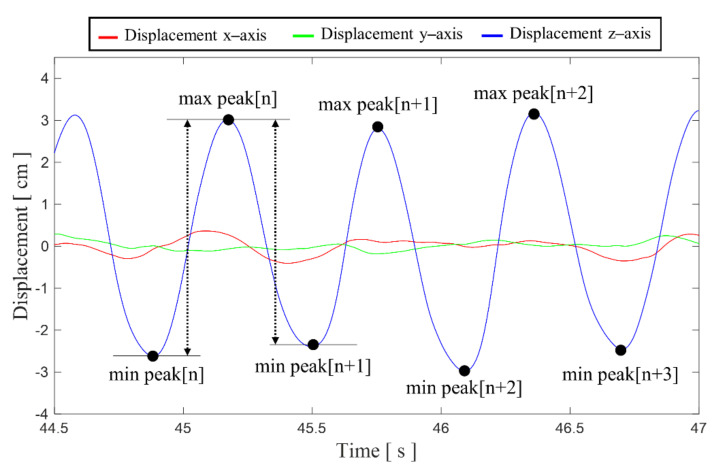
Example of min and max peaks on the displacement waveform for [*n*], [*n* + 1], [*n* + 2]th chest compression.

For real-time feedback, the in-house software calculates displacement by chest compression in real-time based on measurement of min peak, max peak, and second min peak. As an example, Figure 4 shows min, max, and second min peak on the *z*-axis displacement waveform. Using Equation (5), the distance of CC for each axis is calculated as the values of these three peaks. Finally, the CCD is calculated using a vector sum of distances of three axes, as shown in Equation (6).

(5) $$d_{x}\left[n\right]=\left\{\left(\left|max\;peak\left[n\right]-min\;peak\left[n\right]\right|\right)+\left(\left|max\;peak\left[n\right]-min\;peak\left[n+1\right]\right|\right)\right\}/2$$

(6) $$d\left[n\right]=\sqrt{d_{x}{\left[n\right]}^{2}+d_{y}{\left[n\right]}^{2}+d_{z}{\left[n\right]}^{2}}$$

When providing continuous CCD feedback, smooth measures are more important than immediate feedback. We computed CCD for feedback by averaging the depths of five consecutive compressions (Equation (7)).

(7) $$CCD\_feedback\left[n\right]=\left\{\begin{array}{c} \frac{\sum_{m=1}^{n}d\left[m\right]}{n}\;\;\;if\;n<5\\ \frac{\sum_{m=n-4}^{n}d\left[m\right]}{5}\;\;\;if\;n\geq 5 \end{array}\right.$$

### 2.3. Experimental Settings to Evaluate the Accuracy of the Proposed Depth Feedback System

**Figure 5:**
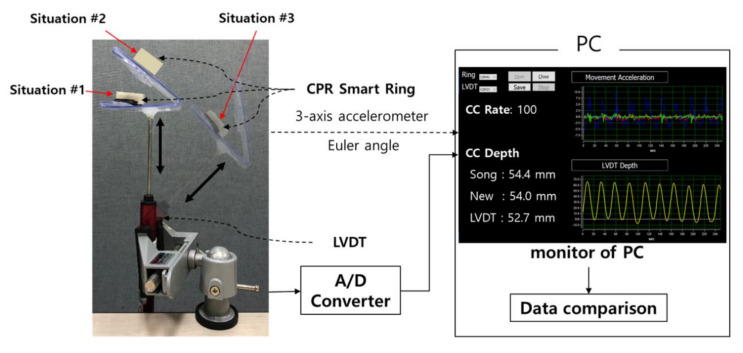
Experimental settings to evaluate the accuracy of the proposed depth feedback system.

We set up an experiment to evaluate the accuracy of the proposed smart-ring-based CCD feedback system, as shown in Figure 5. The CPR Smart Ring transmits the calculated Euler angle value and raw three-axis acceleration data to the PC via Bluetooth. The baud rate was 115,200 and the sampling rate was 100 Hz.

To verify CCD algorithm accuracy, a linear variable differential transformer (LVDT, model RDP-100S; Radian Co., Ltd., Seoul, Korea) that converts change of distance into voltage was used as a reference. This LVDT could measure from 0 to 10 cm, and it was calibrated with a linear two-point method based on the linearity of LVDT output. We acquired the LVDT data using an STM evaluation kit (STM32F4 Discovery, STMicroelectronics, Geneva, Switzerland) and set the sampling rate to 100 Hz, the same as that of the CPR Smart Ring system.

The monitoring software was programmed with Visual Studio (Microsoft Co., Redmond, WA, USA) to simultaneously receive data from the smart ring system and the LVDT. The monitoring software also includes the previous algorithm by Song et al. [10] for accuracy comparison and a new depth estimation algorithm. 

As shown in Equation (8), the error of the smart ring estimation algorithm determines the difference between the reference (LVDT) data and the depth estimated using the smart ring system. The results were derived by calculating the mean and standard deviation (SD) of error.

(8) $$Error\left[n\right]=\left|\;Reference\left[n\right]-CCD\_feedback\left[n\right]\;\right|$$

We conducted two experiments to evaluate the accuracy of the proposed depth feedback system. While observing the depth via a PC monitor, we engaged in up-and-down movements to simulate CC during CPR.

**Figure 6:**
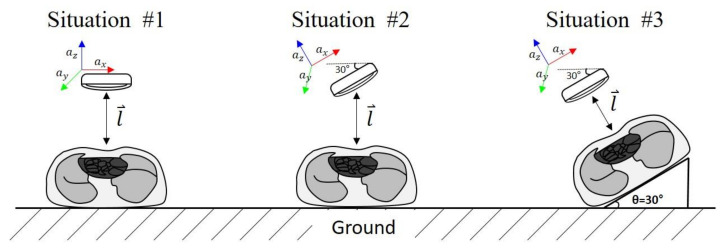
Geometric configurations for the experiments.

The first experiment evaluated various depths (21–30, 31–40, 41–50, and 51–60 mm) with compression performed in the direction of gravity, as in situation #1 in Figure 6. The second experiment compared the accuracy of the system in three situations, as shown in Figure 6, at a fixed CC depth (51–60 mm). Situations #1 and #2 assumed chest compressions in the direction of gravity based on the angle of the smart ring. Situation #3 was a rare case simulating compressing parallel to the body at a 30° angle to the direction of gravity.

During these two experiments, the compression rate was fixed using a metronome sound played at a rate of 100 ticks/min. For data acquisition, the first experiment was repeated for five sets of 100 compressions at each depth, and the second experiment was repeated for five sets of 100 compressions at each situation.

### 2.4. CPR Pilot Test Using an Adult/Infant Mannequin

A pilot test was conducted to compare the accuracy of CC depth estimated using the smart ring and a smartwatch, with the depth of the LVDT of the mannequin used as a reference. Four emergency medical professionals with actual CPR experience in adults and infants participated in this experiment.

The four participants wore both a smart ring and a smartwatch and performed CCs on an adult mannequin (SkillReporter Resusci Anne^®^, Laerdal, Stavanger, Norway) and on an infant mannequin (Resusci Baby QCPR^®^, Laerdal, Stavanger, Norway). To minimize the effect of fatigue, the experiments were separated by sufficient rest intervals (1 h). The CC experiment on an adult mannequin was performed with the smart ring on the index finger and a target compression depth of 5.5 cm. The experiment on the infant mannequin was performed for both the two-finger and two-thumb CC methods. In the two-finger CC method, the middle of the infant’s chest was vertically pressed with the index and middle fingers while the smart ring was worn on the index finger. The two-thumb CC method was performed with the two thumbs, one equipped with a smart ring, and covering the entire chest of the infant with the two hands. In the three mannequin pilot tests, chest compressions were performed at a rate of 100 per minute for 2 min at a predetermined target compression depth, and the compression rate was guided using a metronome. Agreement with target compression depth was ensured by measuring the compression depth of the mannequin via an associated laptop. The smartwatch applied the same algorithm as the smart ring.

## 3. Results

We describe the results in two parts. The results described in Section 3.1 are for the accuracy evaluation of the developed ring-based CCD feedback system. Song et al. and the proposed CCD algorithm were compared with (1) various depth ranges in situation #1, and (2) depth in situation #1–3. In Section 3.2, we describe the results of the CPR pilot test using the adult/infant mannequin wearing the developed ring-based system and smartwatch. 

### 3.1. Accuracy of Estimated Depth of the Ring-Based CCD Feedback System

Table 1 shows the mean and SD of the error, which is the absolute difference between LVDT (reference) depth and each algorithm’s depth, as shown in Equation (8), at each depth range (21–30, 31–40, 41–50, and 51–60 mm) in situation #1 for both the proposed CCD estimation algorithm and the algorithm of Song et al. The proposed algorithm showed a slightly better value than the algorithm of Song et al.

**Figure 7:**
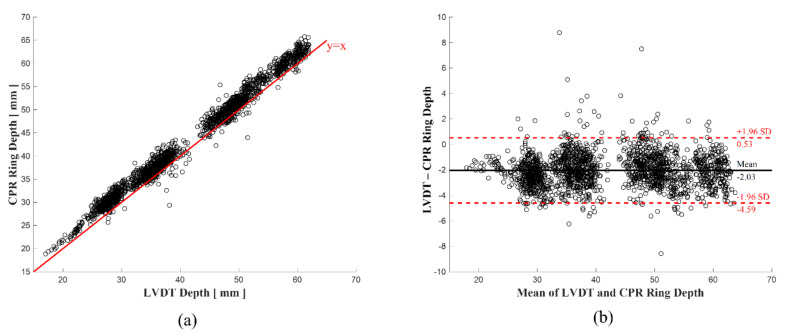
Comparison of reference (LVDT) and feedback algorithms. (**a**) Scatter plot, (**b**) Bland–Altman Plot.

To compare the total data over a depth of 21–60 mm, Figure 7a shows a scatterplot of LVDT and the CPR Smart Ring data. Most of the data are above the y = x line, indicating that the CPR Smart Ring device overestimated depth. Figure 7b uses the Bland-Altman plot method to compare the LVDT and CPR Smart Ring depth measurements based on the mean and SD of the data differences. If the difference is centered on the mean, the CPR Smart Ring depth accuracy is high compared to the reference.

**Figure 8:**
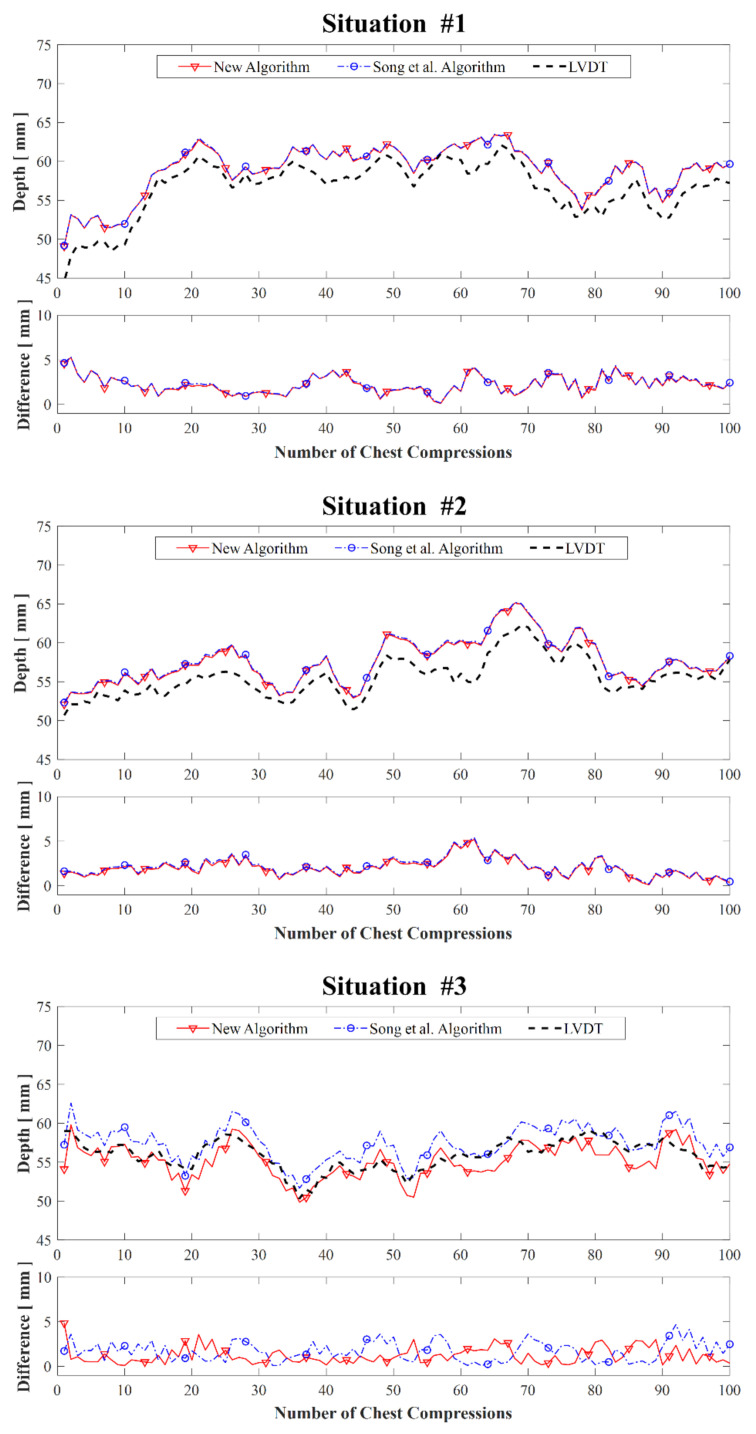
Comparison of depth and error for each situation shown in Figure 6.

Table 2 shows the mean and SD of the error (Equation (8)) for the three situations with 500 aggregated measurements (one per compression). In situations #1 and #2, the Song et al. algorithm and the proposed algorithm provided results with similar values. However, in situation #3, the proposed algorithm outperformed the Song et al. algorithm. Figure 8 shows the real-time values for depth and error for each situation, demonstrating the tendency of each algorithm in the three situations and the performance difference of the algorithms.

The compression rate was based on a metronome in all experiments, so there was no significant deviation from the rate of 100 compressions/min. For Experiment 1, the mean and SD of compression rate of the proposed and Song et al. algorithms were 101 ± 2 compressions/min, and the mean and SD of LVDT (reference) were 100 ± 1 compressions/min. For the compression rate in Experiment 2, the mean and SD of the proposed and Song et al. algorithms were both 101 ± 1 compressions/min, and the mean and SD of the LVDT (reference) were 101 ± 1 compressions/min.

### 3.2. Accuracy in the CPR Pilot Test with Mannequins

**Figure 9:**
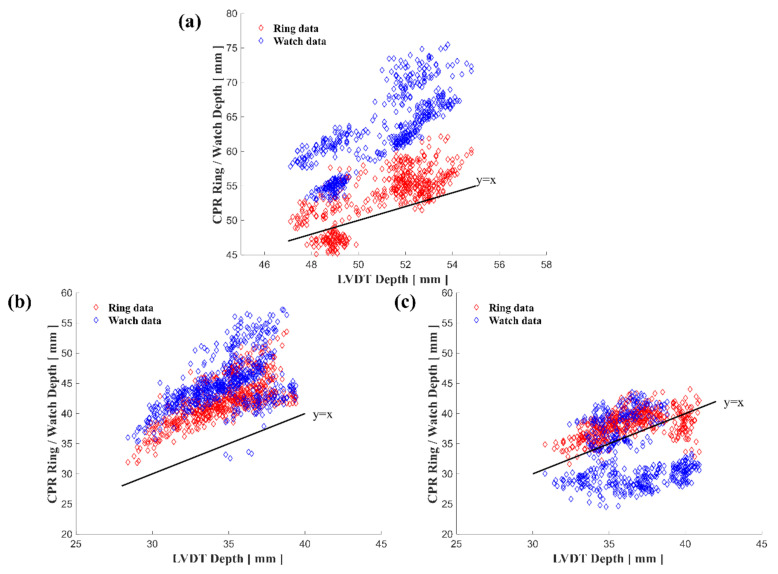
Comparison of mannequin LVDT depth and ring and watch depth. (**a**) Adult mannequin experiment. (**b**) Infant mannequin experiment using the two-finger method. (**c**) Infant mannequin experiment using the two-thumb method.

The accuracy of the smart ring and smartwatch was tested with CPR experiments on both an adult and an infant mannequin. Figure 9 shows a comparison of the estimated depth data of the smart ring and smartwatch to depth data of the mannequin LVDT. In Figure 9, the data count was 400 for each device, with the data estimating the depth for each of 100 chest compressions performed by four emergency medical professionals. 

For the estimated CPR depth of the smart ring, the error results with the adult mannequin (3.1 ± 1.9 mm) and the two-thumb method on the infant mannequin (2.9 ± 1.8 mm) were similar to those of the estimated depth of ring-based CCD feedback system (1.9 ± 1.1 mm). For the estimated CPR depth of the smartwatch, there was a large difference for adult mannequin LVDT and for the two-thumb method on the infant mannequin. The results of the two-finger method on the infant mannequin were overestimated by both the smart ring and the smartwatch. The overall data patterns in the three CPR experiments showed lower mean and SD in the smart ring data than in the smartwatch data (Table 3).

## 4. Discussion

To the best of our knowledge, the smart-ring-based CC feedback device is the first attempt at creating a smart ring CCD system. In this study, we described a smart-ring-based CC feedback device that includes CCD feedback for high-quality CPR. For feedback evaluation, we analyzed the accuracy at various depths, grasping orientations, and compression directions. As a result, the mean of the errors was approximately 2 mm, in the acceptable range for a CCD feedback device. Furthermore, the new algorithm was slightly more accurate than the previous. In situation #3, there was greater difference between the new and Song et al. algorithms. This difference is attributed to movement in a direction other than that of gravity, which is not removed by the Song et al. algorithm. 

Three mannequin pilot experiments (two-hand CC method for adults, two-finger CC method for infants, and two-thumb CC method for infants) were conducted using the smart-ring-based CC feedback device. On the adult mannequin, the proposed smart ring device showed more accurate results than the smartwatch. However, the CC estimated was larger than the reference value provided by the mannequin LVDT, which indicates that the wrist was overly relaxed during chest relaxation. For the two-finger CC method with the infant mannequin, both the ring and watch values were overestimated. The index and middle fingers were bent during chest compression, producing overestimated values due to unnecessary movements. For the two-thumb CC method with the infant mannequin, the estimated depth of the ring showed results similar to those of the mannequin LVDT. However, it was difficult to trust the estimated depth of the watch, whose movement direction differed from the direction of the chest compression, with the wrist moving in a direction close to horizontal during chest compression. Two CPR pilot tests on the mannequins, including the two-hand CC method for adults and the two-thumb CC method for infant, confirmed that the smart ring can be used to estimate CC depth. Comparing the two-finger and the two-thumb CC methods for infants, several studies have reported that the two-thumb CC method is more effective [18,19]. The reason is that the two-finger method is difficult to fix the CPR posture compared to the two-thumb method. Hence, we recommend the two-thumb CC method rather than the two-finger method for accurate CCD feedback when performing infant CPR using the developed ring-based CCD feedback system.

CCD feedback devices using acceleration sensors have inherent limitations. First, accelerometer-based CCD calculations are strongly affected by motion artifacts. However, since CPR is a periodic motion with a short-time period (100 compressions/min), the effect of motion artifacts can be minimized [20]. Second, the CCD feedback device cannot detect sufficient recoil. This limitation occurs with most feedback devices based on one accelerometer. Therefore, the user must maintain recoil when using an accelerometer-based CCD feedback device. Thirdly, when the patient is lying on a soft surface, the CC feedback device based on the accelerometer can overestimate the CCD by including compression of the surface [21]. However, several methods can solve this problem. Use of a backboard can reduce surface compression [22], and there is a method of overcompressing, considering that the feedback is the sum of the depth at which the mattress is compressed and the depth at which the patient’s chest is compressed [23,24]. Oh et al. suggested a dual accelerometer system in which one accelerometer was placed on the patient’s sternum and the other between the patient’s back and the surface [25]. In this system, the difference in distance between the accelerometers is calculated as the actual CCD. This method is expected to provide accurate feedback when the patient is lying on a soft surface and could be applied by pairing a smartphone positioned between the patient’s back and the mattress and a smart ring on the CPR provider’s finger.

There is not yet certainty about the effectiveness of the CCD feedback device for patients. Studies have reported the development and usefulness of CC feedback devices [26,27,28,29,30]. However, according to Kirkbright et al. [31], in both mannequin and human studies, while feedback during resuscitation can produce CC parameters closer to recommended values, there is no evidence that this translates into improved patient outcomes. The reason for this has not been elucidated, and further patient-centered research is warranted. Verification of clinical efficacy is needed for application of a CC feedback device. Moreover, there are very few studies on CC feedback devices for infants [14,15]. Previous studies used the two-finger method with a smartwatch or prototype CPR device that would be difficult to use in real CPR situations. The developed ring-based feedback system is more accurate and more comfortable to wear than previous studies, so it will be useful for real infant CPR situations.

The developed CPR Smart Ring can provide an indication for agreement with target depth in red (insufficient CCD), green (adequate CCD), and blue (excessive CCD) colors produced by a mounted light emitting diode (LED). Considering that CCR is important for high-quality chest compressions, it is guided based on 100 LED flashes per minute. We could extract the CCR from the algorithm, but we thought that it would be better to adopt the guide method due to the structural limitations of the CPR Smart Ring. Several studies have suggested that metronome guides help maintain CCR [32]. However, it is difficult to listen to a metronome in noisy situations, while a flashing light could more helpful [33].

It is expected that the proposed smart-ring-based CCD feedback device will provide more accurate feedback than a smartphone- or a smartwatch-based device. Park et al. [34] concluded that error varies depending on attachment location when using a smartphone as a CCD feedback device; that is, the closer the CCD feedback device is to the patient’s sternum, the more accurate the feedback. The smart-ring-based CCD feedback device is positioned close to the patient’s sternum to provide accurate feedback, though it is less popular than a smartphone or a smartwatch. In addition, since the developed smart ring system is relatively light, convenient to move, and highly cost-effective, it can be expected to be used in CPR education and training.

## 5. Conclusions

As a proof-of-concept, this study verified the usefulness of the proposed smart-ring-based CCD feedback device in CPR situations through various experiments. Through the CPR pilot tests with mannequins, it was shown to be applicable to real CPR situations. However, clinical trials must be conducted in a variety of situations before using it in the field. As a future study, we will conduct a feasibility test of the proposed CCD feedback system through an adult/infant mannequin experiment with a large sample.

## Figures and Tables

**Table 1:** Absolute errors at the four compression depths.

Absolute Errors of Compression Depth (mm)								
	21–30 mm		31–40 mm		41–50 mm		51–60 mm	
	Mean	SD	Mean	SD	Mean	SD	Mean	SD	
**Proposed Algorithm**	2.41	0.95	1.99	1.12	1.92	1.12	1.99	1.10	
**Song et al. Algorithm**	2.59	0.96	2.14	1.15	2.10	1.17	2.08	1.11	

**Table 2:** Absolute errors in the three measurement situations shown in Figure 6.

Absolute Error (mm)						
	Situation #1		Situation #2		Situation #3	
	Mean	SD	Mean	SD	Mean	SD
**Proposed Algorithm**	1.99	1.10	2.19	0.88	1.36	1.09
**Song et al. Algorithm**	2.08	1.11	2.32	0.89	2.03	1.41

**Table 3:** Absolute errors in two positions of compression depth.

Absolute Error between Either the Smart Ring or Smartwatch and the Mannequin LVDT (mm)						
	Adult Mannequin		Infant Mannequin (Two Finger)		Infant Mannequin (Two Thumb)	
	Mean	SD	Mean	SD	Mean	SD
**|Mannequin LVDT—Ring|**	3.1	1.9	7.6	2.4	2.9	1.8
**|Mannequin LVDT—Watch|**	11.8	4.2	10.7	3.5	7.7	5.3

## Data Availability

Data sharing is not applicable to this article.
